# Catastrophic cognitions about coronavirus: the Oxford psychological investigation of coronavirus questionnaire [TOPIC-Q]

**DOI:** 10.1017/S0033291721000283

**Published:** 2021-01-22

**Authors:** Laina Rosebrock, Emma Černis, Sinéad Lambe, Felicity Waite, Stephanie Rek, Ariane Petit, Anke Ehlers, David M. Clark, Daniel Freeman

**Affiliations:** 1 Department of Psychiatry, University of Oxford, Oxford, UK; 2 Oxford Health NHS Foundation Trust, Oxford, UK; 3 Department of Psychology, Ludwig-Maximilians-University of Munich, Munich, Germany; 4 International Max Planck Research School for Translational Psychiatry (IMPRS-TP), Munich, Germany; 5 Department of Experimental Psychology, University of Oxford, Oxford, UK

**Keywords:** cognitions, coronavirus, COVID-19, factor analysis, cognitive therapy, mental health, stress

## Abstract

**Background:**

Cognitive therapies are developed on the principle that specific cognitive appraisals are key determinants in the development and maintenance of mental health disorders. It is likely that particular appraisals of the coronavirus pandemic will have explanatory power for subsequent mental health outcomes in the general public. To enable testing of this hypothesis we developed a questionnaire assessing coronavirus-related cognitions.

**Methods:**

12 285 participants completed online a 46-item pool of cognitions about coronavirus and six measures of different mental health problems. The sample was randomly split into derivation and validation samples. Exploratory factor analyses determined the factor structure, selection of items, and model fit in the derivation sample. Confirmatory factor analysis (CFA) then tested this model in the validation sample. Associations of the questionnaire with mental health outcomes were examined.

**Results:**

The 26-item, seven-factor, Oxford Psychological Investigation of Coronavirus Questionnaire [TOPIC-Q] was developed. CFA demonstrated a good model fit (χ^2^ = 2108.43, df = 278, *p* < 0.001, comparative fit index (CFI) = 0.950, Tucker−Lewis index (TLI) = 0.942, root mean square error of approximation (RMSEA) = 0.033, standardized root mean square residual (SRMR) = 0.038). The factors were: cognitions about (1) safety and vulnerability, (2) negative long-term impact, (3) having the virus, (4) spreading the virus, (5) social judgment, (6) negative self, and (7) being targeted. The questionnaire explained significant variance in depression (45.8%), social anxiety (37.3%), agoraphobia (23.2%), paranoia (27.3%), post-traumatic stress disorder (57.1%), and panic disorder (31.4%). Cognitions about negative long-term impact had the greatest explanatory power across disorders.

**Conclusions:**

TOPIC-Q provides a method to assess appraisals of the pandemic, which is likely to prove helpful both in longitudinal studies assessing mental health outcomes and in delivery of psychological therapy.

## Introduction

The coronavirus pandemic has had an impact on mental health across the globe (Pierce et al., 2020; Sonderskov, Dinesen, Santini, & Ostergaard, 2020; Wang et al., 2020; Zhu et al., 2020). There has been – at least in the short-term – a deterioration in wellbeing and increased rates of anxiety, depression, and posttraumatic stress (Vindegaard & Benros, 2020). The longer-term effects on mental health remain to be seen. The reason for the expectation of an increase in mental health difficulties is that the pandemic is clearly a stressor, which may, for example, bring financial difficulties, inactivity, isolation, and health fears. This leads to multiple related questions: Why does the same stressor lead to different psychological effects? What are the drivers of poorer mental health outcomes at the individual level of explanation? What might clinicians need to target when patients present in services? In this paper we prepare the ground to study the importance of appraisals of the pandemic.

A key determinant of reactions to the pandemic is likely to be what people think. This is one of the central insights of cognitive therapy: psychological disorders are characterized by cognitions of differing content. For example, thinking that the self is worthless and the future is hopeless is fundamental to depression (Beck, Rush, Shaw, & Emery, 1979), misinterpreting anxiety symptoms as signs of a heart attack can drive panic attacks (Clark, 1986), and appraising a stressor as one's own fault and meaning that the world is completely unsafe keeps the sense of threat underlying post-traumatic stress disorder (PTSD) persisting (Ehlers & Clark, 2000). One recent survey found that higher perceived severity of COVID-19 (e.g. how severe participants believed the infection and death rate to be) and reduced perceptions of self-control were associated with overall mental health problems (e.g. an increase in negative emotions and poor sleep) in the Chinese public (Li, Yang, Dou, & Cheung, 2020), providing preliminary support for an association between cognitions about the pandemic and wellbeing. We set out to develop a comprehensive self-report assessment of cognitions about the coronavirus pandemic that may account for a number of common adverse mental health outcomes.

We focussed on six mental health conditions. We assessed specific appraisals of the pandemic that may contribute to the occurrence of depression (e.g. ‘My life is worthless now’), social anxiety (e.g. ‘Other people will think I'm horrible if I get close to them’), agoraphobia (e.g. ‘I will die if I leave the house’), paranoia (e.g. ‘People are deliberately trying to give me the virus’), and PTSD (e.g. ‘The world is no longer safe,’ ‘My response shows I am a bad person’). Further, we assessed appraisals of the nature of the virus itself, including the meaning of shortness of breath and fever, which may exacerbate panic attacks (e.g. ‘Every time I struggle to breathe, I think I'm dying’). In addition to disorder-specific cognitions, there are likely to be shared cognitive themes across the disorders that contribute to mental health outcomes (e.g. beliefs about one's ability to cope and the long-term impact of consequences resulting from the pandemic). The aims of the study were to (1) develop a measure of potentially modifiable cognitions related to the coronavirus pandemic and lockdown and (2) determine whether specific cognitions are particularly associated with specific mental health outcomes. It was expected that catastrophic cognitions about coronavirus would be associated with each mental health outcome to some degree.

## Method

### Participants

Participants were adults (18 years or older) living in the United Kingdom. Participants were recruited over a two-week period (15th–29th May 2020) via advertisements on Facebook. The advertisement text was: ‘We would like adults in the UK to complete this online survey, which will take approximately 30 min. The aim of the survey is to understand the psychological factors that may lead to mental health difficulties in the wake of the coronavirus epidemic. This information will be used to provide better psychological treatment.’ Participants completed an online survey presented using Qualtrics (www.qualtrics.com). In all, 12 285 participants completed the new cognitions questionnaire. Ethical approval was obtained from the Medical Sciences Inter-Divisional Research Ethics Committee (IDREC) at the University of Oxford (R69638) and all procedures contributing to the study complied with the ethical standards of the relevant committees on human experimentation and with the Helsinki Declaration.

### Measures

#### TOPIC Questionnaire (TOPIC-Q):

An item pool comprising 46 cognitions was developed by the authors (clinical psychologists with expertise in cognitive models of mental health disorders). The item content drew on the definitions in cognitive models of the key cognitions for each of the six disorders assessed in this study and also for two additional conditions (health anxiety and obsessive−compulsive disorder). Each item was rated using a 5-point response scale asking about current degree of endorsement (0 = not at all, 1 = a little, 2 = moderately, 3 = a lot, 4 = totally). A full list of the items can be found in Table 1.

**Table 1.** TOPIC Questionnaire – original item pool

1. I'm in great danger of catching coronavirus if I leave my home.
2. I'm in great danger of spreading the virus if I leave my home.
3. The only way to survive is not to leave the house.
4. People will think I'm infected with coronavirus if I cough or sneeze in public.
5. People will judge me badly because of my response to coronavirus.
6. People will think I'm disgusting if I cough or sneeze in public.
7. People will think I'm horrible if I get too close to them.
8. I am going to die from this virus.
9. The people I love are going to die from this virus.
10. People have let me down during the crisis.
11. I should have done more to help during the crisis.
12. I cannot forgive the government for risking my life through their response to the virus.
13. I will be financially ruined.
14. The virus will destroy everything I care about.
15. My response to the pandemic shows I am inadequate.
16. My world has been shattered by coronavirus.
17. I will never be safe from the virus.
18. Having to isolate has permanently changed me for the worse.
19. My response to the lockdown shows that I am a bad person.
20. My life is worthless now.
21. Whenever my breath is short I think I've got the virus.
22. If I feel hot, I think I'm dying.
23. If I cough, I'm certain I have the virus.
24. If others tell me I look tired, I fear I have the virus.
25. I have coronavirus but no one has detected it.
26. Testing negative for coronavirus wouldn't reassure me.
27. If I get coronavirus, no treatment will save me.
28. I am especially physically vulnerable to coronavirus.
29. The virus is on almost every surface.
30. Everything is contaminated with the virus.
31. I have spread the virus to hundreds of people.
32. I have spread the virus and caused other people to die.
33. I have spread the virus without realizing I had it.
34. People are deliberately trying to give me the virus.
35. The virus is particularly going after me.
36. The virus was manufactured to target me and the people close to me.
37. When outside, people get close to me in order to give me the virus.
38. If anyone is going to get coronavirus, it will definitely be me.
39. I deserve to get coronavirus.
40. There's nothing I can do to prevent myself from catching the virus.
41. Nobody would care if I died from coronavirus.
42. I have failed in my response to coronavirus.
43. The pandemic has made everything hopeless.
44. There is no point planning ahead.
45. If people get close to me, I worry that I will become aggressive with them.
46. When I'm outside, I worry that other people will become aggressive with me.

### Mental health outcome measures

**Patient Health Questionnaire-9** (PHQ-9; Kroenke, Spitzer, & Williams, 2001): The PHQ-9 is a 9-item self-report questionnaire measuring symptoms of depression over the last two weeks. Each of the items corresponds to a diagnostic criteria item for major depression. The response scale ranges from 0 (not at all) to 3 (nearly every day). Higher scores indicate higher levels of depression. Scores of 10 and above indicate clinically elevated levels of depression. The PHQ-9 in this sample showed high internal consistency (Cronbach's alpha = 0.92).

**Social Phobia Inventory** (SPIN; Connor et al., 2000): The SPIN is a 17-item self-report measure of social anxiety over the last week. One item was modified slightly to account for social distancing restrictions (‘I avoid going to parties’ was changed to ‘I avoid going to parties or online parties’). The response scale ranges from 0 (not at all) to 4 (extremely). Higher scores indicate higher levels of social anxiety. The clinical cut-off is a score of 19 or above. The SPIN in this sample showed high internal consistency (Cronbach's alpha = 0.94).

**Mobility Inventory for Agoraphobia** (MI; Chambless, Caputo, Jasin, Gracely, & Williams, 1985): The MI is a self-report measure assessing current avoidance of situations due to anxiety (i.e. agoraphobia) when alone. Items ask about avoidance of places (e.g. restaurants, car parks), transportation (e.g. buses, trains), riding/driving in a car, and specific situations (e.g. walking on the street, standing in lines). The response scale ranges from 1 (never avoid) to 5 (always avoid) and there is an option to select ‘N/A.’ The average score is derived from all items that were not answered N/A. Higher average scores indicate higher levels of agoraphobia. An average score of 2.3 and above is considered the clinical cut-off. The MI in this sample showed high internal consistency (Cronbach's alpha = 0.92).

**Revised Green et al., Paranoid Thoughts Scale – Persecution subscale** (R-GPTS; Freeman et al., 2019): The R-GPTS persecution subscale is a 10-item self-report questionnaire assessing thoughts of being persecuted over the last two weeks. The response scale ranges from 0 (not at all) to 4 (totally). Higher scores indicate higher levels of paranoia. Scores of 11 and above indicate moderately severe levels of persecutory beliefs. The R-GPTS in this sample showed high internal consistency (Cronbach's alpha = 0.93).

**PTSD Checklist for DSM-5** (PCL-5; Weathers et al., 2013): The PCL-5 is a 20-item self-report measure that assesses the DSM-5 symptoms of PTSD in relation to the most distressing experience participants had during lockdown. In the introduction to this questionnaire, we included examples of traumas (e.g. losing loved ones, being admitted to hospital) as well as other stressful consequences of the pandemic (e.g. significant financial worries, seeing difficult images on TV) and asked participants to answer questions with their most distressing experience in mind. PTSD symptoms were measured over the past two weeks. The response scale ranges from 0 (not at all) to 4 (extremely). Higher scores indicate greater severity of PTSD symptoms. A score of 33 and above is considered the clinical cut-off for PTSD (Bovin et al., 2016). The PCL in this sample showed high internal consistency (Cronbach's alpha = 0.95).

**Panic Disorder Severity Scale** (PDSS; Shear et al., 1997): The PDSS is a 7-item self-report measure of panic symptoms over the past week. The items assess frequency of panic attacks, worry about future panic attacks, impact on daily activities, and avoidance of situations that may lead to panic attacks occurring. The item response scale ranges from 0 to 4. Higher scores indicate greater symptom severity for panic disorder. Scores of 8 and above are considered the clinical cut-off. The PDSS in this sample showed high internal consistency (Cronbach's alpha = 0.90).

### Analyses

Factor analyses were conducted in R, version 4.0.0 (R Core Team, 2020) with packages psych (version 1.8.12; Revelle, 2019) and lavaan (version 0.6–3.1295; Rosseel, 2019). For deriving the final set of items, the sample was split using *R*'s random sampling function into a derivation (*n* = 6142) and validation (*n* = 6143) sample. In the derivation sample, exploratory factor analyses (EFA) using principal axis factoring and varimax rotation were carried out to assess the structure of items and refine the item pool by deleting poor-fitting items. Varimax rotation was chosen due to low-to-moderate correlations among items. Prior to conducting EFA, the pairwise item correlations were examined and items that were either poorly correlated with all other items or had very high correlations (above 0.80, indicating multicollinearity) were deleted. Items were then considered for deletion 1–2 at a time during EFA based on factor loadings (not loading higher than 0.30 on any factor, or loadings above 0.30 on more than one factor), communalities (<0.30), and content of items (e.g. theoretically inconsistent or redundant). The number of factors to extract was determined through parallel analysis and examination of the scree plot.

The final measurement model was then re-assessed in the validation sample using confirmatory factor analysis (CFA) with MLR robust maximum-likelihood estimator. Model fit was assessed using a comparative fit index (CFI) and Tucker−Lewis index (TLI) of >0.95, a root mean square error of approximation (RMSEA) of <0.06, and a standardized root mean square residual (SRMR) of <0.08 (Hu & Bentler, 1999). Modification indices were used to help identify the best-fitting model.

The associations between the factors of TOPIC-Q and mental health outcomes were examined using correlations and structural equation modelling (SEM). Analyses were also conducted in *R*. The factor scores for each TOPIC-Q factor were correlated with each mental health outcome score (PHQ-9, SPIN, MI, R-GPTS, PCL-5, and PDSS) using Pearson's *r*. Six models were then assessed (one for each mental health outcome) using SEM. SEM comprises a CFA and a structural model, which estimates the relations among constructs (Kline, 2015). For each of the six mental health outcomes, all TOPIC-Q factor scores were entered into the model to determine the overall fit and the relative contribution of each of the factors (i.e. types of coronavirus cognitions) to the mental health outcome score. We utilized the MLR robust maximum-likelihood estimator. (As an additional sensitivity analysis for the PCL-5, the cognitive items (‘having strong negative beliefs about yourself, other people, or the world’ and ‘blaming yourself or someone else for the stressful experience or what happened after it’) were removed for repetitions of the correlations and SEM analyses to avoid a potential confound with the content of TOPIC-Q. The results were unchanged and therefore the analyses with the full PCL are reported.).

Criterion validity was established by testing differences (using *t* tests) for three variables likely to increase endorsement of catastrophic cognitions: whether someone close to the participant had died from COVID-19; whether the participant was at high clinical risk for a severe course of COVID-19 (i.e. had cardiovascular disease, was immunocompromised, had liver/kidney disease, current/previous cancer, or significant cigarette consumption); and whether the participant had received a mental health diagnosis. We also ran correlations of each TOPIC-Q factor with age given an association between age and decreased mental health problems (e.g. Jorm, 2000). Factor scores for each TOPIC-Q factor were also used for all criterion validity analyses.

## Results

### Participant characteristics

The participant characteristics are summarized in Table 2. The mean age of the sample was 54.6 years (s.d. = 14.8; range: 18–91). Participants were predominantly female and White British. Over half of the participants were married (or in a civil partnership) or cohabiting. Over half were employed part or full-time or were self-employed.

**Table 2.** Participant characteristics

	*n*	%
Gender		
Male	3310	26.9
Female	8864	72.2
Other/Prefer not to say	111	0.9
Ethnicity		
White British	11 009	89.6
White Other	820	6.7
White and Black Caribbean/Black African/Asian	86	0.7
Other mixed ethnic background	76	0.6
Indian/Pakistani/Bangladeshi	72	0.6
Chinese	21	0.2
Any other Asian background	30	0.2
African/Caribbean	17	0.1
Any other Black/African/Caribbean background	4	0.0
Arab	5	0.0
Any other ethnic group/Prefer not to say	145	1.2
Marital status		
Single	2308	18.8
Cohabiting/Married or civil partnership	8007	65.1
Divorced/Separated	1334	10.9
Widowed	636	5.2
Employment status (pre-coronavirus)		
Unemployed	311	2.5
Employed Part/Full-time	5362	43.6
Self-employed	1251	10.2
Retired	3909	31.8
Student	518	4.2
Homemaker	367	3.0
Voluntary	156	1.3
Disabled/Long-term sick leave	411	3.3

Average scores on the mental health questionnaires are provided in Table 3. 27.8% of the sample scored above the clinical cut-off for depression, 32.5% scored above the clinical cut-off for social anxiety, 67.7% scored above the clinical cut-off for agoraphobia, 6.6% reported moderately severe paranoia, 13% scored above the clinical cut-off for PTSD, and 9.8% scored above the clinical cut-off for panic symptoms. The high rate of participants scoring above the clinical cut-off for agoraphobia is likely due to the lockdown measures and potentially the mean age of the sample (i.e. a significant proportion of the participant group would have been at higher risk of developing a severe course of COVID-19 and therefore may have been more cautious about going outside).

**Table 3.** Clinical characteristics

	*n*	Mean	s.d.
Mental health outcomes			
Depression (PHQ-9)	10 850	7.05	6.43
Social anxiety (SPIN)	11 137	15.36	13.89
Agoraphobia (MI)	11 218	2.90	1.13
Paranoia (R-GPTS)	10 974	2.29	5.37
PTSD symptoms (PCL-5)	10 640	14.61	15.01
Panic symptoms (PDSS)	11 046	1.82	4.12

Note: PHQ-9 = Patient Health Questionnaire – 9, SPIN = Social Phobia Inventory, MI = Mobility Inventory for Agoraphobia, R-GPTS = Revised Green et al., Paranoid Thoughts Scale, PCL-5 = PTSD Checklist for DSM-5, PDSS = Panic Disorder Severity Scale.

### Development of TOPIC-Q

Factor analysis was appropriate as Bartlett's test of Sphericity was significant (χ^2^ = 127 762.6, df = 1035, *p* < 0.001) and the Kaiser−Myer−Olkin test of sampling adequacy was high (KMO = 0.94).

Following the criteria for removing poor-fitting items, EFA using the development sample (*n* = 6142) led to 20 of the original 46 items being discarded. One item (‘Everything is contaminated with the virus’) was deleted prior to EFA due to being strongly correlated with another similarly worded item. During EFA, an additional 19 items were deleted: nine items had communalities below 0.3 (‘People have let me down during the crisis,’ ‘I should have done more to help during the crisis,’ ‘I cannot forgive the government for risking my life through their response to the virus,’ ‘I will be financially ruined,’ ‘I have coronavirus but no one has detected it,’ ‘Testing negative for coronavirus wouldn't reassure me,’ ‘The virus was manufactured to target me and the people close to me,’ ‘There's nothing I can do to prevent myself from catching the virus,’ ‘If people get close to me, I worry that I will become aggressive with them’); six items strongly cross-loaded over multiple factors (‘I'm in great danger of catching coronavirus if I leave my home,’ ‘The virus will destroy everything I care about,’ ‘My response to the pandemic shows I am inadequate,’ ‘My life is worthless now,’ ‘If anyone is going to get coronavirus, it will definitely be me,’ ‘Nobody would care if I died from coronavirus’); two items no longer loaded onto any factors after the removal of cross-loading items (‘I'm in great danger of spreading the virus if I leave my home’, ‘When I'm outside, I worry that other people will become aggressive with me’). Two items were deleted after modification indices were run due to strongly correlated residuals (‘The people I love are going to die from this virus,’ ‘I am especially physically vulnerable to coronavirus’).

The scree plot and parallel analysis test of the remaining 26 items indicated that a seven-factor model was the most appropriate fit for the data. This model explained 55.9% of the variance.

A CFA with the validation sample (*n* = 6143) was run using the seven-factor 26-item model from EFA. This indicated a good model fit (χ^2^ = 2108.43, df = 278, *p* < 0.001, CFI = 0.950, TLI = 0.942, RMSEA = 0.033, SRMR = 0.038). Factor loadings of the final items are presented in Table 4. Factors were identified as ‘Cognitions about Safety and Vulnerability’, ‘Cognitions about Negative Long-Term Impact’, ‘Cognitions about Having the Virus’, ‘Cognitions about Negative Self’, ‘Cognitions about Social Judgment’, ‘Cognitions about Spreading the Virus’, and ‘Cognitions about Being Targeted’.

**Table 4.** Final items and factor loadings from exploratory factor analysis (EFA) and confirmatory factor analysis (CFA)

TOPIC-Q Factors	EFA loadings	CFA loadings
**Cognitions about Safety and Vulnerability**		
3. The only way to survive is not to leave the house.	0.576	0.606
8. I am going to die from this virus.	0.702	0.761
17. I will never be safe from the virus.	0.618	0.746
27. If I get coronavirus, no treatment will save me.	0.671	0.727
29. The virus is on almost every surface.	0.472	0.612
**Cognitions about Negative Long-Term Impact**		
16. My world has been shattered by coronavirus.	0.650	0.747
18. Having to isolate has permanently changed me for the worse.	0.600	0.733
43. The pandemic has made everything hopeless.	0.787	0.845
44. There is no point planning ahead.	0.638	0.718
**Cognitions about Having the Virus**		
21. Whenever my breath is short I think I've got the virus.	0.705	0.812
22. If I feel hot, I think I'm dying.	0.758	0.860
23. If I cough, I'm certain I have the virus.	0.781	0.850
24. If others tell me I look tired, I fear I have the virus.	0.660	0.790
**Cognitions about Negative Self**		
19. My response to the lockdown shows that I am a bad person.	0.693	0.722
39. I deserve to get coronavirus.	0.472	0.564
42. I have failed in my response to coronavirus.	0.704	0.787
**Cognitions about Social Judgment**		
4. People will think I'm infected with coronavirus if I cough or sneeze in public.	0.700	0.768
5. People will judge me badly because of my response to coronavirus.	0.444	0.501
6. People will think I'm disgusting if I cough or sneeze in public.	0.837	0.840
7. People will think I'm horrible if I get too close to them.	0.522	0.620
**Cognitions about Spreading the Virus**		
31. I have spread the virus to hundreds of people.	0.862	0.841
32. I have spread the virus and caused other people to die.	0.765	0.836
33. I have spread the virus without realizing I had it.	0.598	0.657
**Cognitions about Being Targeted**		
34. People are deliberately trying to give me the virus.	0.724	0.715
35. The virus is particularly going after me.	0.572	0.607
37. When outside, people get close to me in order to give me the virus.	0.673	0.722

The TOPIC-Q factors in this sample all showed good internal consistency: Cognitions about Safety and Vulnerability (Cronbach's alpha = 0.82), Cognitions about Negative Long-Term Impact (Cronbach's alpha = 0.84), Cognitions about Having the Virus (Cronbach's alpha = 0.89), Cognitions about Negative Self (Cronbach's alpha = 0.73), Cognitions about Social Judgment (Cronbach's alpha = 0.77), Cognitions about Spreading the Virus (Cronbach's alpha = 0.74), and Cognitions about Being Targeted (Cronbach's alpha = 0.73). The final questionnaire can be found in the appendix (Table A1).

### Associations with mental health symptoms

All TOPIC-Q factors were significantly correlated with each of the outcome measures (see Table 5). Depression and social anxiety were most strongly correlated with Cognitions about Negative Long-Term Impact and Cognitions about Negative Self. Social anxiety was also strongly associated with Cognitions about Having the Virus, Cognitions about Social Judgment, and Cognitions about Safety and Vulnerability. Agoraphobia was principally associated with Cognitions about Safety and Vulnerability. Paranoia was most strongly associated with Cognitions about Being Targeted, Cognitions about Negative Self and Cognitions about Negative Long-Term Impact. PTSD was most strongly correlated with Cognitions about Negative Long-Term Impact. Lastly, panic disorder symptoms were most strongly correlated with Cognitions about Having the Virus, Cognitions about Negative Long-Term Impact and Cognitions about Negative Self.

**Table 5.** Correlations between TOPIC-Q factors and mental health outcomes

TOPIC-Q factors	PHQ-9 total (*n* = 10 850)	SPIN total (*n* = 11 137)	MI total (*n* = 11 218)	R-GPTS total (*n* = 10 974)	PCL-5 total (*n* = 10 640)	PDSS total (*n* = 11 046)
Safety and Vulnerability	0.41*	0.43*	0.44*	0.26*	0.52*	0.39*
Negative Long-Term Impact	0.62*	0.51*	0.26*	0.39*	0.70*	0.47*
Having the Virus	0.42*	0.45*	0.28*	0.28*	0.53*	0.45*
Social Judgment	0.38*	0.43*	0.28*	0.28*	0.45*	0.32*
Negative Self	0.52*	0.50*	0.18*	0.41*	0.56*	0.45*
Spreading the Virus	0.27*	0.28*	0.08*	0.25*	0.32*	0.25*
Being Targeted	0.35*	0.38*	0.21*	0.40*	0.44*	0.38*

Note: PHQ-9 = Patient Health Questionnaire – 9, SPIN = Social Phobia Inventory, MI = Mobility Inventory for Agoraphobia, R-GPTS = Revised Green et al., Paranoid Thoughts Scale, PCL-5 = PTSD Checklist for DSM-5, PDSS = Panic Disorder Severity Scale.

*p < 0.001.

The results of the SEMs are reported in Table 6. Coronavirus cognitions explained 45.8% of the variance in depression scores (χ^2^ = 3739.27, df = 297, *p* < 0.001, CFI = 0.951, TLI = 0.942, RMSEA = 0.033, SRMR = 0.037), 37.3% of the variance in social anxiety scores (χ^2^ = 3782.75, df = 297, *p* < 0.001, CFI = 0.951, TLI = 0.943, RMSEA = 0.032, SRMR = 0.036), 23.2% of the variance in agoraphobia scores (χ^2^ = 3943.69, df = 297, *p* < 0.001, CFI = 0.950, TLI = 0.941, RMSEA = 0.033, SRMR = 0.036), 27.3% of the variance in paranoia scores (χ^2^ = 3598.71, df = 297, *p* < 0.001, CFI = 0.951, TLI = 0.942, RMSEA = 0.032, SRMR = 0.036), 57.1% of the variance in PTSD symptom scores (χ^2^ = 3600.55, df = 297, *p* < 0.001, CFI = 0.953, TLI = 0.944, RMSEA = 0.032, SRMR = 0.036), and 31.4% of the variance in panic symptoms (χ^2^ = 3662.75, df = 297, *p* < 0.001, CFI = 0.951, TLI = 0.943, RMSEA = 0.032, SRMR = 0.036).

**Table 6.** SEM analyses for each TOPIC-Q factor

	Standardized *β*	*z* value	*p*
**Depression (PHQ-9)**			
SafeVul	−0.050	−3.11	**0.002**
LTImpact	0.522	30.56	**<0.001**
HaveVir	0.060	4.24	**<0.001**
SocJudge	0.060	5.07	**<0.001**
NegSelf	0.177	9.69	**<0.001**
VirSpread	0.031	2.60	**0.009**
Target	−0.024	−1.55	0.122
**Social anxiety (SPIN)**			
SafeVul	0.023	1.38	0.168
LTImpact	0.205	12.28	**<0.001**
HaveVir	0.108	7.17	**<0.001**
SocJudge	0.166	13.51	**<0.001**
NegSelf	0.239	12.35	**<0.001**
VirSpread	0.009	0.72	0.473
Target	0.055	3.14	**0.002**
**Agoraphobia (MI)**			
SafeVul	0.512	29.64	**<0.001**
LTImpact	−0.062	−4.28	**<0.001**
HaveVir	−0.033	−2.61	**0.009**
SocJudge	0.037	2.89	**0.004**
NegSelf	0.042	3.00	**0.003**
VirSpread	−0.003	−0.29	0.773
Target	−0.029	−2.43	**0.015**
**Paranoia (R-GPTS)**			
SafeVul	−0.124	−6.43	**<0.001**
LTImpact	0.201	9.36	**<0.001**
HaveVir	−0.025	−1.27	0.205
SocJudge	0.107	7.97	**<0.001**
NegSelf	0.173	6.49	**<0.001**
VirSpread	0.034	2.07	**0.038**
Target	0.285	9.89	**<0.001**
**PTSD symptoms (PCL-5)**			
SafeVul	0.002	0.128	0.898
LTImpact	0.535	31.63	**<0.001**
HaveVir	0.134	10.04	**<0.001**
SocJudge	0.061	5.79	**<0.001**
NegSelf	0.115	7.09	**<0.001**
VirSpread	0.045	3.86	**<0.001**
Target	0.027	1.86	0.063
**Panic symptoms (PDSS)**			
SafeVul	−0.014	−0.72	0.470
LTImpact	0.221	10.96	**<0.001**
HaveVir	0.215	10.42	**<0.001**
SocJudge	0.033	2.54	**0.011**
NegSelf	0.170	6.96	**<0.001**
VirSpread	0.000	−0.02	0.984
Target	0.088	3.82	**<0.001**

Note: PHQ-9 = Patient Health Questionnaire – 9, SPIN = Social Phobia Inventory, MI = Mobility Inventory for Agoraphobia, R-GPTS = Revised Green et al., Paranoid Thoughts Scale, PCL-5 = PTSD Checklist for DSM-5, PDSS = Panic Disorder Severity Scale. SafeVul = Cognitions about Safety and Vulnerability, LTImpact = Cognitions about Negative Long-Term Impact, HaveVir = Cognitions about Having the Virus, SocJudge = Cognitions about Social Judgment, NegSelf = Cognitions about Negative Self, VirSpread = Cognitions about Spreading the Virus, Target = Cognitions about Being Targeted.

Most factors significantly contributed to the variance in scores across disorders, but there were specific factors which accounted for scores within each disorder to a higher degree: Cognitions about Negative Long-Term Impact most strongly accounted for depression scores and PTSD. Cognitions about Negative Self, Negative Long-Term Impact, and Social Judgment most strongly accounted for symptoms of social anxiety. Cognitions about Safety and Vulnerability most strongly accounted for agoraphobia. Cognitions about Being Targeted, Negative Long-Term Impact, and Negative Self most strongly accounted for symptoms of paranoia. Lastly, Cognitions about Having the Virus, Negative Long-Term Impact and Negative Self most strongly accounted for panic symptoms.

### Criterion validity

The results from the *t* tests are summarized in Table 7. Nearly all of the TOPIC-Q factors were more strongly endorsed if the participant had a close friend or family member die from COVID-19, had physical health problems that put them at high risk for a severe COVID-19 illness, or had a mental health diagnosis. The only exception was that participants who were at higher physical health risk rated Cognitions about Spreading the Virus as lower, which might be understood as this group adhering to social distancing/shielding guidelines to a greater extent. Furthermore, there were no differences in Cognitions about Negative Self between those who were high risk and those who were not. Lastly, nearly all of the TOPIC-Q factors were negatively associated with age, such that older age was associated with lower endorsement of Cognitions about Negative Long-Term Impact (*r* = −0.20, *p* < 0.001), Cognitions about Having the Virus (*r* = −0.16, *p* < 0.001), Cognitions about Social Judgment (*r* = −0.15, *p* < 0.001), Cognitions about Negative Self (*r* = −0.25, *p* < 0.001), Cognitions about Spreading the Virus (*r* = −0.25, *p* < 0.001), and Cognitions about Being Targeted (*r* = −0.11, *p* < 0.001). The only exception was for Cognitions about Safety and Vulnerability which showed a small but significant positive association (*r* = 0.03, *p* < 0.001), and again may show awareness of age increasing physical health risk.

**Table 7.** *t* tests comparing TOPIC-Q factors for relevant variables to establish criterion validity

		Cognitions about Safety and Vulnerability		Cognitions about Negative Long-Tterm Impact		Cognitions about Having the Virus		Cognitions about Social Judgment		Cognitions about Negative Self		Cognitions about Spreading the Virus		Cognitions about Being Targeted	
	*n*	Mean (s.d.)	*t*	Mean (s.d.)	*t*	Mean (s.d.)	*t*	Mean (s.d.)	*t*	Mean (s.d.)	*t*	Mean (s.d.)	*t*	Mean (s.d.)	*t*
Someone close died															
No	10 681	−0.02 (0.62)	−5.38**	−0.02 (0.72)	−4.42**	−0.02 (0.67)	−3.21*	−0.02 (0.76)	−4.02**	−0.01 (0.35)	−3.23*	−0.01 (0.29)	−5.04**	−0.01 (0.21)	−3.24*
Yes	569	0.14 (0.70)		0.14 (0.82)		0.09 (0.79)		0.12 (0.80)		0.05 (0.43)		0.08 (0.44)		0.03 (0.29)	
High risk															
No	7062	−0.12 (0.56)	−24.10**	−0.04 (0.71)	−7.61**	−0.04 (0.65)	−6.73**	−0.06 (0.75)	−8.86**	−0.003 (0.36)	−0.68	0.01 (0.32)	5.44**	−0.01 (0.20)	−7.48**
Yes	4465	0.18 (0.69)		0.06 (0.77)		0.05 (0.73)		0.08 (0.79)		0.002 (0.37)		−0.02 (0.28)		0.02 (0.25)	
Mental health diagnosis															
No	6100	−0.13 (0.54)	−22.07**	−0.17 (0.60)	−26.08**	−0.14 (0.51)	−22.21**	−0.12 (0.72)	−17.20**	−0.08 (0.23)	−23.46**	−0.03 (0.25)	−11.70**	−0.03 (0.14)	−16.53**
Yes	5427	0.13 (0.70)		0.18 (0.83)		0.15 (0.82)		0.13 (0.80)		0.08 (0.46)		0.03 (0.35)		0.04 (0.29)	

*p < 0.05, **p < 0.001

## Discussion

Cognitive appraisals are key determinants of mental health and wellbeing (Beck, 1970). Understanding cognitions about the current pandemic could therefore provide useful information about the types of mental health problems likely to be seen in services once the immediate threat of the pandemic subsides. This study could inform the delivery of cognitive therapy, a recommended treatment for each of the mental health outcomes measured in this study. We therefore set out to develop the first measure of catastrophic cognitions about coronavirus. Factor analyses indicated that the final seven-factor 26-item measure was robust with a good model fit. Importantly, the way people think about the coronavirus pandemic is associated in understandable ways with each of the chosen mental health outcomes.

As expected, coronavirus cognitions contributed to a large amount of variance in each of the mental health outcomes. Furthermore, certain types of cognitions more strongly explained variance in scores in certain disorders. Cognitions about Negative Long-Term Impact was the strongest contributor to depression, PTSD, and panic disorder, and the second strongest contributor to social anxiety after Cognitions about Negative Self. How people think about the persistence of negative consequences of the pandemic looks to be an important factor in understanding psychological reactions. Unsurprisingly, Cognitions about Safety and Vulnerability was the strongest contributor to agoraphobia while Cognitions about Being Targeted most strongly contributed to paranoia. Finally, Cognitions about Having the Virus strongly contributed to panic symptoms, suggesting that catastrophic appraisals about symptoms of the virus (e.g. shortness of breath, fever) may exacerbate panic attacks. TOPIC-Q may be a useful tool in assessing these types of cognitions in clinical services to aid in the use of existing evidence-based therapies (e.g. cognitive behavioural techniques; Hofmann, Asnaani, Vonk, Sawyer, & Fang, 2012). The questionnaire also demonstrated criterion validity; factor scores were higher for those who had lost someone to COVID-19, were physically at high risk, and had received a mental health diagnosis.

There are several key limitations to note. First, although this was a large sample, it was not representative of the population (e.g. the sample was predominantly female, White, and older). Due to the recruitment method (e.g. Facebook advertisements), there will have been a selection bias. It also comprised only UK residents, limiting generalizability to other countries. Further studies are needed to examine these cognitions in other groups. Second, we did not carry out an evaluation of test−retest reliability. Third, although the items were developed with expertise from clinical psychologists specializing in cognitive theories of mental health disorders, we did not obtain direct patient and public involvement. Lastly, we cannot make causal inferences due to the cross-sectional nature of the design. Longitudinal and interventionist studies are needed to clarify the causal impact of the cognitions on long-term mental health outcomes, particularly as restrictions ease. TOPIC-Q should facilitate such studies.

Unfortunately it is likely that the psychological consequences of the coronavirus pandemic will persist for a lengthy duration, and therefore TOPIC-Q will remain a relevant assessment. However, the types of cognitions identified are also likely to prove useful in conceptualizing reactions to future pandemics if they occur.

## Appendix: TOPIC Questionnaire.

**Table A1.** How strongly do you **currently** believe each of these statements?

	Not at all	A little	Moderately	A lot	Totally
1. The only way to survive is not to leave the house.	0	1	2	3	4
2. I am going to die from this virus.	0	1	2	3	4
3. I will never be safe from the virus.	0	1	2	3	4
4. If I get coronavirus, no treatment will save me.	0	1	2	3	4
5. The virus is on almost every surface.	0	1	2	3	4
6. My world has been shattered by coronavirus.	0	1	2	3	4
7. Having to isolate has permanently changed me for the worse.	0	1	2	3	4
8. The pandemic has made everything hopeless.	0	1	2	3	4
9. There is no point planning ahead.	0	1	2	3	4
10. Whenever my breath is short I think I've got the virus.	0	1	2	3	4
11. If I feel hot, I think I'm dying.	0	1	2	3	4
12. If I cough, I'm certain I have the virus.	0	1	2	3	4
13. If others tell me I look tired, I fear I have the virus.	0	1	2	3	4
14. My response to the lockdown shows that I am a bad person.	0	1	2	3	4
15. I deserve to get coronavirus.	0	1	2	3	4
16. I have failed in my response to coronavirus.	0	1	2	3	4
17. People will think I'm infected with coronavirus if I cough or sneeze in public.	0	1	2	3	4
18. People will judge me badly because of my response to coronavirus.	0	1	2	3	4
19. People will think I'm disgusting if I cough or sneeze in public.	0	1	2	3	4
20. People will think I'm horrible if I get too close to them.	0	1	2	3	4
21. I have spread the virus to hundreds of people.	0	1	2	3	4
22. I have spread the virus and caused other people to die.	0	1	2	3	4
23. I have spread the virus without realizing I had it.	0	1	2	3	4
24. People are deliberately trying to give me the virus.	0	1	2	3	4
25. The virus is particularly going after me.	0	1	2	3	4
26. When outside, people get close to me in order to give me the virus.	0	1	2	3	4
